# Hepatitis B infection status among South Africans attending public health facilities over a five-year period: 2015 to 2019

**DOI:** 10.1371/journal.pgph.0000992

**Published:** 2023-09-25

**Authors:** Shelina Moonsamy, Pavitra Pillay, Nishi Prabdial-Sing

**Affiliations:** 1 Division of the National Health Laboratory Service, National Institute for Communicable Diseases, Centre for Vaccines and Immunology, Johannesburg, South Africa; 2 Faculty of Health Sciences, Department of Biomedical and Clinical Technology, Durban University of Technology, Durban, South Africa; 3 Faculty of Health Sciences, Department of Medical Virology, School of Pathology, University of the Witwatersrand, Johannesburg, South Africa; University of Michigan, UNITED STATES

## Abstract

Hepatitis B, a potentially life-threatening viral infection of the liver, remains a global public health concern despite the availability of effective vaccines for over three decades. The aim of our study was to provide national data on active hepatitis B infections in the public health sector of South Africa. We conducted retrospective analyses on national laboratory data over the period 2015 to 2019. We identified 176,530 cases who tested positive for HBsAg (active infection) with a test positivity rate of 9.02%. Of these active infections, 11,355 (6.43%) were found to be chronically infected. We linked 24,839 (14.07%) and 2,461 (21.67%) HBeAg positive results to all active HBV infections and identified chronic infections respectively. Clearance of HBsAg was observed in 5,569 cases, inclusive of clearance in 135 chronic cases. Active HBV infections were significantly higher in men than women over the five years (p < 0.0001). Among individuals who were vaccine-eligible as infants (0 to 19 years old), we observed 4,981 active HBV infections, including 1,131 infections under five years old, majority of which (65.78%) were under one year old. In the under five-year age group, the HBsAg population positivity rate was 0.02% and test positivity rate was 4.83%. Among all women with active HBV infections (78,935), 85.17% were of reproductive age and of these, 13.73% were HBeAg positive. Without a birth dose of the HBV vaccine, lack of routine HBsAg screening at antenatal care, and HBsAg and HBeAg prevalence among women of reproductive age, it is likely that the majority of cases under five years old were vertically infected. Optimal HBV vaccine coverage, inclusive of a birth dose, is key to eliminating horizontal and vertical transmission of HBV. Early identification of HBV chronicity through real time data analysis is fundamental in reducing the risk of liver cirrhosis and hepatocellular carcinoma.

## 1 Introduction

Hepatitis B, a potentially life-threatening viral infection of the liver, remains a global public health concern despite the availability of effective vaccines for over three decades [1–3]. It occurs following infection with the hepatitis B virus (HBV) and common transmission routes are sexual contact, intravenous drug use and vertical transmission from mother to child [4]. Infection with HBV can cause acute and chronic disease. The risk of progression to chronicity decreases with age, ranging from around 70–90% among infants less than a year old to 1–10% among individuals over 20 years old [5–7]. Resolved HBV infection usually results in hepatitis B immunity that prevents disease following re-exposure, however HBV reactivation or reinfection, as identified by the reappearance of detectable HBsAg levels, has been reported in individuals infected with HIV-1 [8]. Chronically infected individuals have a 15–40% lifetime risk of cirrhosis, liver failure or hepatocellular carcinoma [9,10]. Presently, there is no complete cure for chronic HBV infection as this requires the clearance of HBsAg and all forms of HBV DNA, including intranuclear covalently closed circular DNA (cccDNA) [11,12]. In South Africa, hepatitis B disease progression may be managed with various treatment options as highlighted in the National Guidelines for the Management of Viral Hepatitis [9,10,13–15]. HBsAg clearance may occur at an annual rate of around 1–2% of chronically infected individuals, either naturally or following treatment interventions [13,15–17]. This is considered as an optimal endpoint (functional cure) following antiviral treatment [16,18,19]. In 2019, The World Health Organisation (WHO) estimated that 296 million people were living with chronic HBV infection, a 15% increase from the 2015 estimate of 257 million [20,21]. In June 2016, the World Health Assembly adopted a resolution to eliminate viral hepatitis as a major public health problem by 2030 [1]. Elimination is targeted at reducing new chronic infections by 90% and HBV related deaths by 65% [1]. In addition, one of the WHO impact targets for measuring elimination is ≤0.1% HBsAg prevalence in children under five years old [22].

HBV infection is diagnosed following laboratory detection of HBsAg, which is the first serological biomarker to appear following HBV infection and indicates active infection [23,24]. If HBsAg is not cleared within 6 months of first detection, HBV infection is classified as chronic [1,23,25,26]. The existence of HBsAg indicates that a person is infectious [27]. Transient HBsAg positivity may be observed up to two weeks following HBV vaccination [27,28]. HBeAg appears shortly after HBsAg appearance in acute infection and can be detected in chronic HBV infections as well [24,29]. The presence of HBeAg indicates high levels of viral replication and is a biomarker of increased infectivity [24,29–32]. In South Africa, as per the national guidelines, HBeAg testing follows a positive HBsAg test [15]. Pregnant women who are HBeAg positive are at increased risk of transmitting HBV to their infants [33–35].

Hepatitis B was highly endemic in South Africa before April 1995 when the HBV vaccine was introduced as a monovalent dose into the South African expanded programme on immunisation (EPI) schedule [36]. Since December 2015, the HBV vaccine is one of the vaccines among the hexavalent combination vaccine of diphtheria, tetanus, acellular pertussis, inactivated poliovirus, Haemophilus influenza type B and hepatitis B (DTaP-IPV-HIB-HepB). The current HBV vaccination schedule consists of doses at 6, 10 and 14 weeks of age (primary series), and a booster dose at 18 months of age [37,38]. Although recommended to minimise the risk of mother to child transmission of HBV, a birth dose of the HBV vaccine is yet to be included in the EPI schedule of South Africa [39,40]. Vaccination coverage for South Africa among children under one year old who have completed their primary course of immunisation has been reported from 2000 to 2019 by the Health Systems Trust of South Africa using data obtained from the District Health Information System [41–45]. Over the 20-year period, the average vaccination coverage rate by province ranged from 67.20% in North West to 85.57% in Northern Cape (S1 Table).

In South Africa, chronic HBV prevalence has been estimated at 2.5 million cases, reported in 2014 [6]. Based on the population size of South Africa in 2014 at just over 54 million, this would translate to a HBsAg prevalence of 4.6%, indicative of intermediate HBV endemicity (2 to 7% HBsAg prevalence) [36,46–48]. A major transmission route in South Africa was reported to be unexplainable horizontal transmission in children under five years of age, whilst sexual transmission was reported to be the major transmission route among adolescents and young adults [36]. Over the five years, 2015 to 2019, the average population of South Africa, as reported by Statistics South Africa was just under 57 million [49]. Overall, there were higher numbers of women than men, although in Gauteng and North West, there were slightly more men than women. Provincially, the smallest province of South Africa (Gauteng), housed the majority of South Africans, whilst the largest province (Northern Cape, just over 20-fold larger) housed the least number of South Africans at more than 10-fold lower than Gauteng [50].

Most HBV studies that have been conducted in South Africa targeted distinct cohorts [36,38,39,51–56]. Our first study on prevalence and incidence rates of laboratory-confirmed hepatitis B infection in South Africa, 2015 to 2019 focused on trend analysis of the proportion of HBsAg and anti-HBc IgM positive cases versus all cases tested per marker and per 100,000 population per calendar year [57]. The aim of this study was to determine the proportion of South Africans in the public health sector who had active hepatitis B infection nationally over a five-year period, including children under five years old and women of reproductive age.

## 2 Methods

### 2.1 Ethics statement

Our study was conducted on anonymous retrospective data linked to a unique identifier. Ethics approval was obtained from the Faculty of Health Sciences, Institutional Research Ethics Committee (IREC 069/20) of the Durban University of Technology, Durban, South Africa. Approval to access and analyse the data was obtained via the National Health Laboratory Service (NHLS) Academic Affairs and Research Management System (PR20254).

### 2.2 Study population and data source

We conducted a cross-sectional study on HBsAg and HBeAg tests obtained from the National Health Laboratory Service (NHLS) Central Data Warehouse (CDW) for tests performed nationally during the period 2015 to 2019. The NHLS CDW is the national repository for laboratory data from the public health sector of South Africa, serving over 80% of the country’s population [58]. Records included duplicate entries of a case if the same test was conducted multiple times over the study period. Each entry into the repository was allocated a unique identification number (UID) which was duplicated if a link to an existing record was identified. Criteria for inclusion in our study were records of all valid HBsAg and HBeAg test results.

### 2.3 Data analyses

Data analyses were performed in in Stata/SE (Version 17.0, Texas, USA) and Microsoft Excel (Version 16, USA).

Data were obtained per year from 2015 to 2019 and were appended to maintain two separate datasets; one of all HBsAg test records and the other of all HBeAg test records. Cases were assigned to defined five-year age groups based on the time interval between the sample collection date (first if there were multiple records) and date of birth. All analyses were performed on the appended five-year data files.

Initial analyses were performed to: 1) obtain denominators for HBsAg and HBeAg test positivity rates (line list of total number of cases tested); 2) identify cases with multiple HBsAg tests and their testing patterns; and 3) develop registries of all active HBV infections and chronic HBV infections coupled with HBeAg results. HBsAg records of equivocal (inconclusive) results were maintained initially (for purposes of obtaining denominators) but removed prior to analysis of multiple HBsAg tests as our focus here was solely on positive and negative results. The registries were used to calculate the proportion of cases who were tested for HBeAg over the study period and the proportion with positive HBeAg results. Data were subsequently analysed to determine the distribution of cases by age group and province, both stratified by gender, and to determine the number of active HBV infections per 100,000 province population. We used the average population over the five years as reported by Statistics South Africa [49]. We applied the test of proportions to determine statistical differences (α = 0.05) between women compared to men by age group and by province.

Additional analyses focussed on age group 0 to 4 years and women of reproductive age (15 to 49 years) [50]. Among children 0 to 4 years old, we calculated HBsAg test positivity rate (HBsAg positive cases / total cases 0 to 4 years old tested for HBsAg), HBsAg population positivity rate (HBsAg positive cases / average population size 0 to 4 years old 2015 to 2019), age in one-year intervals, and age in one-week intervals among infants under one year old.

## 3 Results

**Fig 1:**
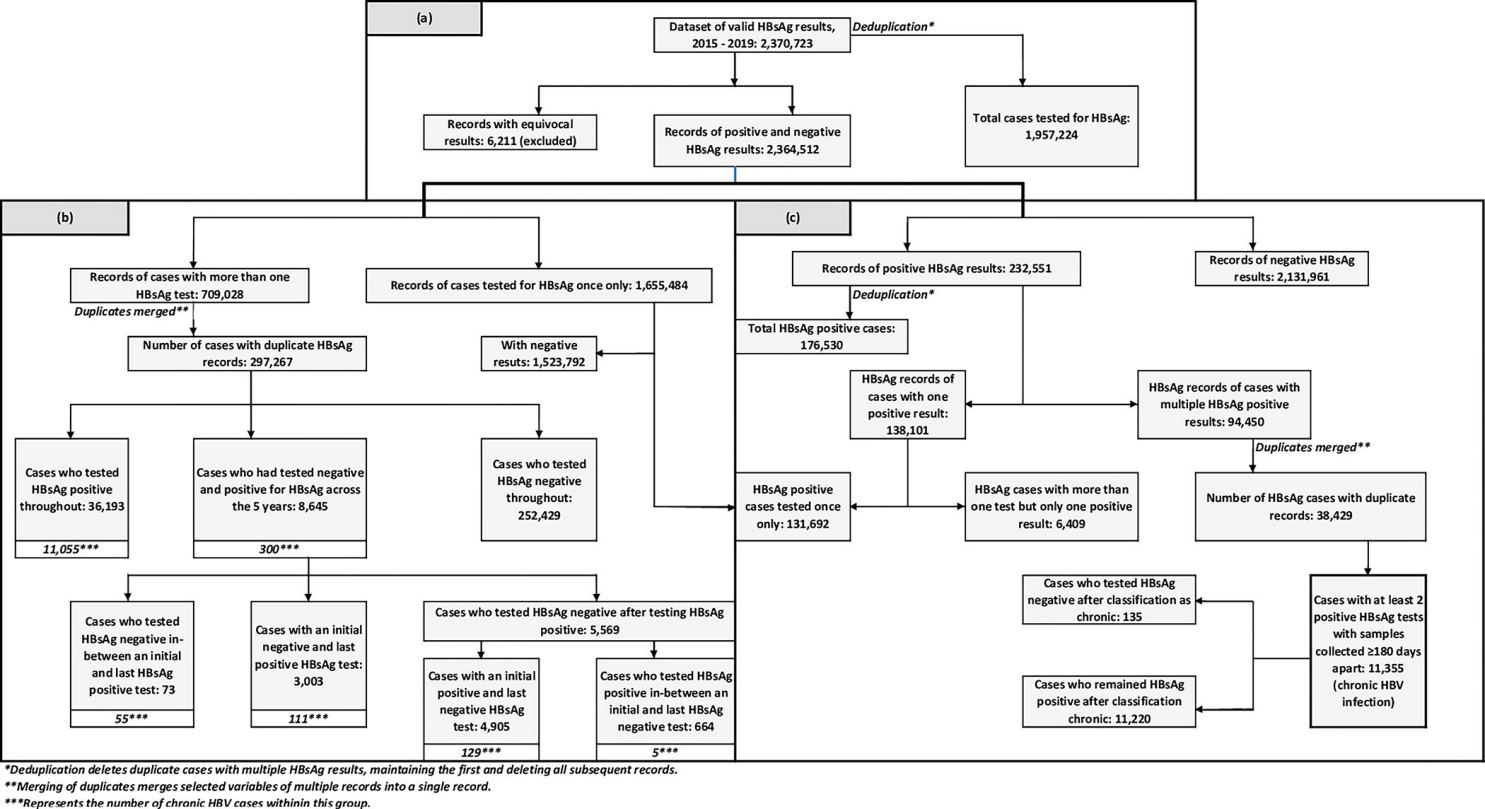
Flow of HBsAg valid records from 2015 to 2019; to obtain a dataset of all positive and negative records (a), to obtain datasets of cases who tested positive throughout, negative throughout, or both positive and negative over the five years (b), and to obtain a dataset of chronic HBV infections (c).

Following development of the appended 2015 to 2019 HBsAg and HBeAg data files, the HBsAg dataset consisted of a total of 2,370,723 records and the HBeAg dataset consisted of a total of 107,433 records [57]. Deduplication of the HBsAg dataset resulted in a line list of 1,957,224 cases (Fig 1A). Deduplication of the HBeAg dataset resulted in a line list of 84,463 cases; 25,796 with positive results (test positivity rate of 30.54%), 58,219 with negative results, and 448 with equivocal results.

From the HBsAg dataset of 2,370,723 records, 6,211 records with equivocal results were removed, leaving 2,364,512 records of positive and negative HBsAg results, of which 1,655,484 cases had been tested once only and the remaining 709,028 cases had at least two tests during the five-year period (Fig 1B). From the 709,028 records, we identified 297,267 cases with duplicate records; 252,429 cases who tested negative for HBsAg throughout, 8,645 cases who had both positive and negative HBsAg results, and 36,193 cases who tested positive for HBsAg throughout (Fig 1B). Of the 8,645 cases who tested positive and negative for HBsAg, 5,569 cases tested negative after a positive test, 3,003 cases tested positive after a negative test, and 73 cases tested negative in between an initial and last positive test (Fig 1B). Of the 5,569 cases who tested negative after a positive test, 4,905 cases tested positive initially and negative subsequently and 664 cases tested positive in between an initial and last negative test (Fig 1B).

### 3.1 Development of a registry and analyses of all active HBV infections coupled with HBeAg results, 2015 to 2019

From the HBsAg dataset of 2,364,512 records, we identified and extracted 232,551 records of positive HBsAg results (Fig 1C). Deduplication of cases resulted in a line list of 176,530 cases who had tested positive for HBsAg at least once during the five-year period (active HBV infection), with a test positivity rate of 9.02% (176,530 / 1,957,224) (Fig 1A and 1C).

**Fig 2:**
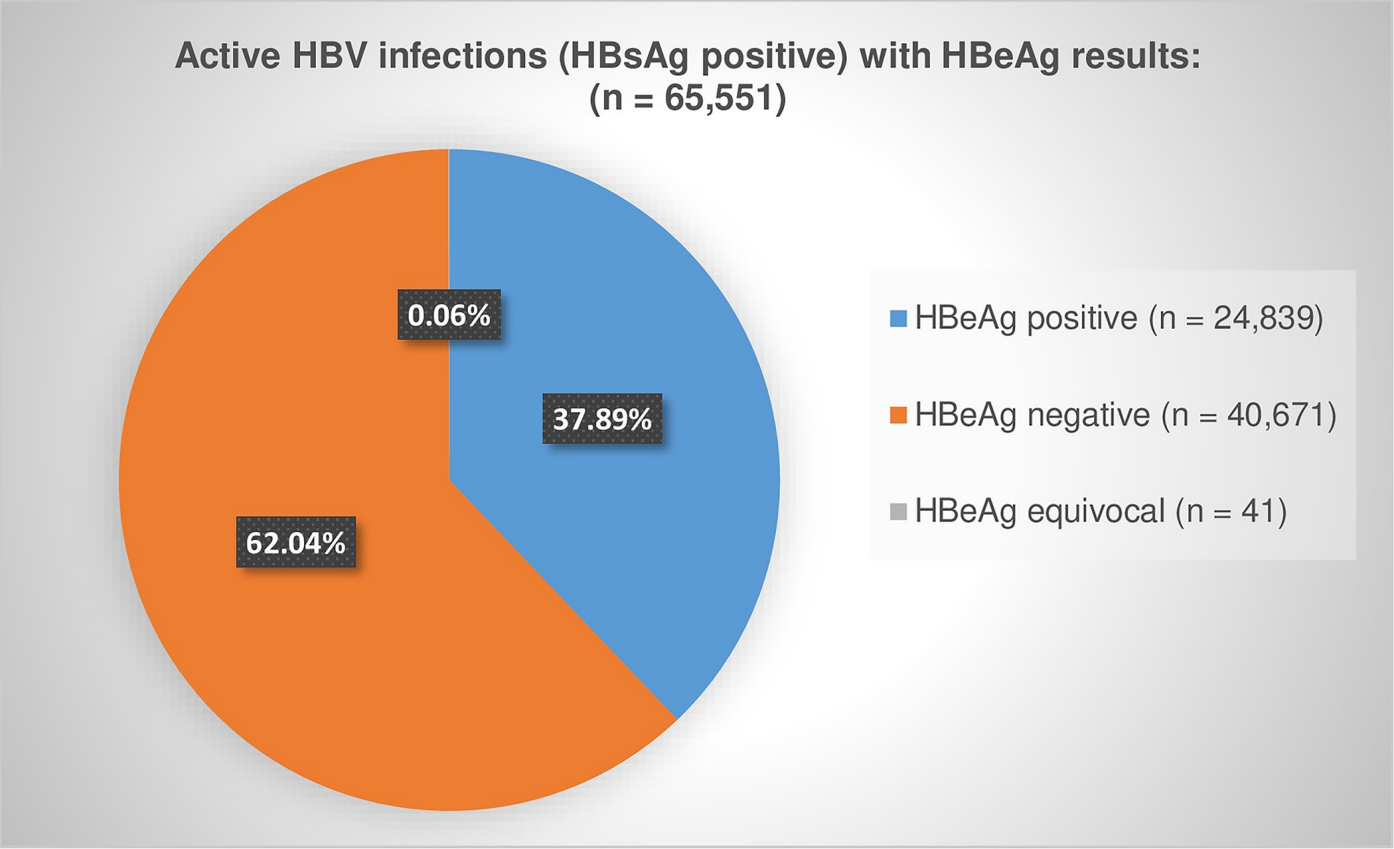
Proportion of active HBV infections with positive, negative and equivocal HBeAg results, 2015 to 2019.

Of the 176,530 active infections, we linked 65,551 (37.13%) cases to a HBeAg result (Table 1). The number of active infections with available HBeAg results per province is shown in Table 2 and actual HBeAg results is illustrated in Fig 2.

**Table 1:** Distribution of active HBV infections by age group stratified by gender and HBeAg results, 2015 to 2019.

Age group	Gender	HBsAg		HBeAg					Age group	Gender	HBsAg		HBeAg				
		Positive		With results^b^		Positive^c^					Positive		With results^b^		Positive^c^		
		No.	*P-value* ^*a*^	No	%	No	%	*P-value* ^*a*^			No.	*P-value* ^*a*^	No	%	No	%	*P-value* ^*a*^
**0–4**	*Women*	502	***0*.*0029***	146	29.08	30	5.98	*0*.*0897*	**40–44**	*Women* ^*d*^	8,152	***<0*.*0001***	2,695	33.06	824	10.11	***<0*.*0001***
	*Men*	603		141	23.38	45	7.46			*Men*	14,720		5,249	35.66	1,900	12.91	
	*Unknown*	26	* *	7	26.92	3	11.54			*Unknown*	312	* * *** ***	138	44.23	43	13.78	* *
	***Total***	**1,131**		**294**	**25.99**	**78**	**6.9**			***Total***	**23,184**		**8,082**	**34.86**	**2,767**	**11.93**	
**5–9**	*Women*	197	*>0*.*9999*	54	27.41	16	8.12	*0*.*3610 *	**45–49**	*Women* ^*d*^	5,182	***<0*.*0001***	1,772	34.2	470	9.07	***<0*.*0001***
	*Men*	196		63	32.14	22	11.22			*Men*	9,631		3,387	35.17	1,159	12.03	
	*Unknown*	14		5	35.71	2	14.29			*Unknown*	196	* * *** ***	97	49.49	33	16.84	
	***Total***	**407**		**122**	**29.98**	**40**	**9.83**			***Total***	**15,009**		**5,256**	**35.02**	**1,662**	**11.07**	
**10–14**	*Women*	394	***<0*.*0001***	127	32.23	50	12.69	*0*.*6244 *	**50–54**	*Women*	3,568	***<0*.*0001***	1,293	36.24	239	6.7	***<0*.*0001***
	*Men*	258		112	43.41	46	17.83			*Men*	5,759		1,992	34.59	538	9.34	
	*Unknown*	12		8	66.67	3	25			*Unknown*	106	* * *** ***	47	44.34	11	10.38	* *
	***Total***	**664**		**247**	**37.2**	**99**	**14.91**			***Total***	**9,433**		**3,332**	**35.32**	**788**	**8.35**	
**15–19**	*Women* ^*d*^	*2*,*017*	***<0*.*0001***	788	39.07	365	18.1	***<0*.*0001 ***	**55–59**	*Women*	2,143	***<0*.*0001***	786	36.68	134	6.25	***<0*.*0001***
	*Men*	722		290	40.17	150	20.78			*Men*	3,494		1,243	35.58	322	9.22	
	*Unknown*	40	* *	22	55	13	32.5			*Unknown*	66	* * *** ***	29	43.94	5	7.58	* * *** ***
	***Total***	**2,779**		**1,100**	**39.58**	**528**	**19**			***Total***	**5,703**		**2,058**	**36.09**	**461**	**8.08**	
**20–24**	*Women* ^*d*^	*8*,*797*	***<0*.*0001***	3,543	40.28	1,659	18.86	***<0*.*0001***	**60+**	*Women*	2,918	***<0*.*0001***	1,145	39.24	185	6.34	***<0*.*0001 ***
	*Men*	4,146		1,721	41.51	890	21.47			*Men*	4,043		1,491	36.88	290	7.17	
	*Unknown*	211	*** ***	126	59.72	58	27.49			*Unknown*	98	* * *** ***	57	58.16	10	10.2	* *
	***Total***	**13,154**		**5,390**	**40.98**	**2,607**	**19.82**			***Total***	**7,059**		**2,693**	**38.15**	**485**	**6.87**	
**25–29**	*Women* ^*d*^	*15*,*365*	***<0*.*0001***	5,817	37.86	2,445	15.91	*0*.*4868 *	**Unknown**	*Women*	1,979	***<0*.*0001***	741	37.44	273	13.79	***<0*.*0001***
	*Men*	11,938		5,020	42.05	2,387	19.99			*Men*	2,858		1,163	40.69	485	16.97	
	*Unknown*	453	* *	242	53.42	112	24.72			*Unknown*	449	* * *** ***	163	36.3	66	14.7	* * *** ***
	***Total***	**27,756**		**11,079**	**39.92**	**4,944**	**17.81**			***Total***	**5,286**		**2,067**	**39.1**	**824**	**15.59**	
**30–34**	*Women* ^*d*^	*15*,*747*	***<0*.*0001***	5,517	35.04	2,068	13.13	***<0*.*0001 ***	**Total**	*Women*	78,935	***<0*.*0001***	28,432	36.02	10,156	12.87	***<0*.*0001***
	*Men*	18,077		7,212	39.9	3,259	18.03			*Men*	94,699		35,751	37.75	14,157	14.95	
	*Unknown*	496	* *	225	45.36	98	19.76			*Unknown*	2,896	* * *** ***	1,368	47.24	526	18.16	
	***Total***	**34,320**		**12,954**	**37.74**	**5,425**	**15.81**			***Total***	**176,530**		**65,551**	**37.13**	**24,839**	**14.07**	
**35–39**	*Women* ^*d*^	11,974	***<0*.*0001***	4,008	33.47	1,398	11.68	***<0*.*0001***									
	*Men*	18,254		6,667	36.52	2,664	14.59										
	*Unknown*	417		202	48.44	69	16.55										
	***Total***	**30,645**		**10,877**	**35.49**	**4,131**	**13.48**										

^a^P-value generated from the test of proportions between women and men, per age group and total

^b^Number and percentage of active HBV infections with available HBeAg results

^c^Number and percentage of active HBV infections with positive HBeAg results

^d^Women of reproductive age (15–49 years).

**Table 2:** Number of active HBV infections with available HBeAg results per province, 2015 to 2019.

	Active HBV infections			
Province	HBsAg+	HBeAg results available		% of HBsAg with HBeAg results available
	No.	No.	% of total HBeAg results	
Eastern Cape	21,280	7,473	11.40	35.12
Free State	8,087	1,967	3.00	24.32
Gauteng	65,085	6,468	9.87	9.94
Kwazulu-Natal	35,532	33,824	51.60	95.19
Limpopo	10,122	819	1.25	8.09
Mpumalanga	14,176	9,999	15.25	70.53
North West	9,540	907	1.38	9.51
Northern Cape	1,827	156	0.24	8.54
Western Cape	10,881	3,938	6.01	36.19
**Total**	**176,530**	**65,551**	**100**	**37.13**

Of the total active HBV infections and those that were HBeAg positive (176,530 and 24,839 respectively), significantly more men tested positive than women (p < 0.0001) (Table 1). Individuals aged 20 to 49 years old had the highest proportion of active infections (144,068, 81,61%) and HBeAg positivity (21,536, 86.70%) (Table 1).

There were 4,981 active HBV infections 0 to 19 years old, of which 1,131 (22.71%) were under five years old (Table 1). In the under five-year age group, the HBsAg test positivity rate was 4.83%, and the HBsAg population positivity rate was 0.02% (Table 3A).

**Table 3:** HBV infection among children under five years old, 2015 to 2019; test positivity and population positivity rates a); age in one-year intervals b), and age in weeks among infants under one year old c).

**(3a)**					
No. of active HBV infections in children under five years old				1,131	
No. of cases under 5 years old tested for HBsAg				23,395	
**HBsAg test positivity rate amongst children under five years old (1,131/23,395)**				**4.83%**	
Average population size amongst children under five years old, 2015 to 2019				5,679,585	
**HBsAg population positivity rate amongst children under five years old (1,131/5,679,585)**				**0.02%**	
**(3b)**					
**Age in years**	**# HBsAg positive cases**				**Percentage of total**
<1	744				65.80%
1	119		387		34.20%
2	92				
3	80				
4	96				
**Total**	**1,131**				**100%**
**(3c)**					
**Age in weeks**		**# HBsAg positive cases / Percentage of total**		**Comments**	
0–5		257 / 34.54		Before administration of the first scheduled HBV vaccine dose at 6 weeks of age	
6–16		345 / 46.37		From initiation of the scheduled primary vaccine series at 6 weeks of age to 2 weeks after the scheduled third dose at 14 weeks of age. Possibility of transient positivity following vaccination	
17–52		142 / 19.09		From three weeks post administration of the scheduled third HBV vaccine dose at 14 weeks of age. Post period of transient positivity following vaccination	
**Total**		**744 / 100**			

Among children under five years old, 65,78% of active infections were in children less than one year old, significantly higher than active infections among children 1 to 4 years old combined (34.22%, p < 0.0001) (Table 3B). Among infants under one year old, 34.54% of cases were 0 to 5 weeks old, 46.37% were 6 to 16 weeks old, and 19.09% were 18 to 52 weeks old (Table 3C).

Among all women with active HBV infection (78,935) and those who were HBeAg positive (10,156), 85.17% (67,234) and 90.87% (9,229) were of reproductive age respectively, a 13.73% HBeAg positivity among women of reproductive age (Table 1).

**Fig 3:**
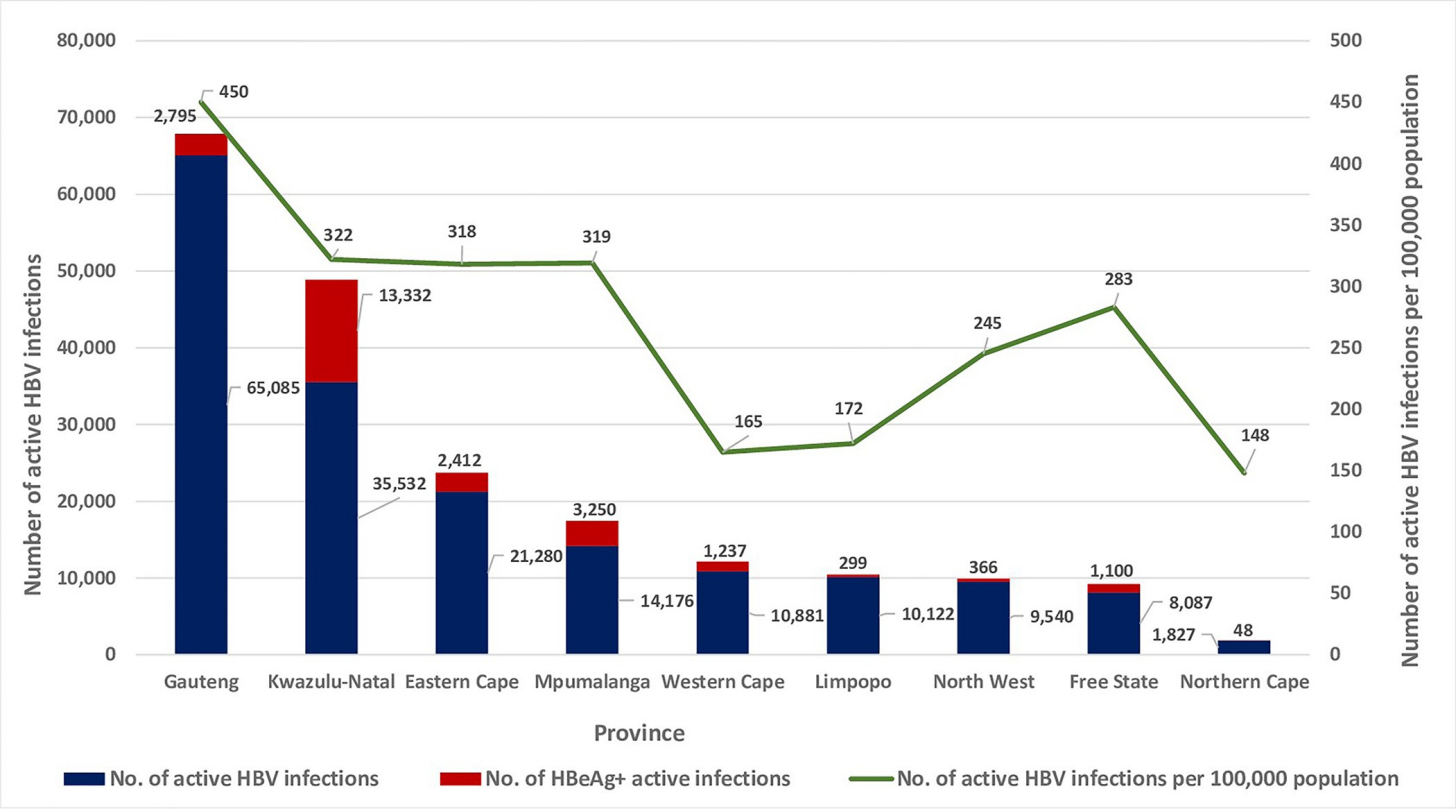
Provincial distribution of active HBV infections, those that were HBeAg positive, and active infections per 100,000 province population, 2015 to 2019.

At a provincial level, of the 176,530 active HBV infections, Gauteng had the highest proportion of cases (65,085, 36.86%), followed by Kwazulu-Natal (35,532, 20.13%) and Eastern Cape (21,280, 12.05%), with the lowest proportion in the Northern Cape (1,827, 1.03%) (Fig 3). Of the 24,839 active infections with HBeAg positive results, individuals from Kwazulu-Natal accounted for more than 50% of the cases (13,332, 53.67%), individuals from Mpumalanga, Gauteng, Eastern Cape, Western Cape and Free State together accounted for 43.46% (10,794) of the cases, and the remaining 2.87% of the cases (713) were from North West, Limpopo and Northern Cape (Fig 3).

The number of active HBV infections per 100,000 population was highest in Gauteng (450/100,000), comparable between Kwazulu-Natal (322/100,000), Mpumalanga (319/100,000) and Eastern Cape (318/100,000), and least in Northern Cape (148/100,000) (Fig 3). In Limpopo, North West and Free State, there was a decline in actual numbers of active HBV infections respectively; however, when we factored in population sizes of these provinces we saw an incline in the number cases respectively (Fig 3).

By gender, the number of active HBV infections in men were significantly higher than women in eight of the nine provinces (p ≤ 0.0306), except for Limpopo with a significantly higher proportion in women (p = 0.0103) (S2 Table). HBeAg positive men were higher than women in all nine provinces, significantly higher in seven of the nine provinces (p ≤ 0.0016), except for Limpopo and Northern Cape. (S2 Table).

### 3.2 Development of a registry and analyses of chronic HBV infections coupled with HBeAg results, 2015 to 2019

**Fig 4:**
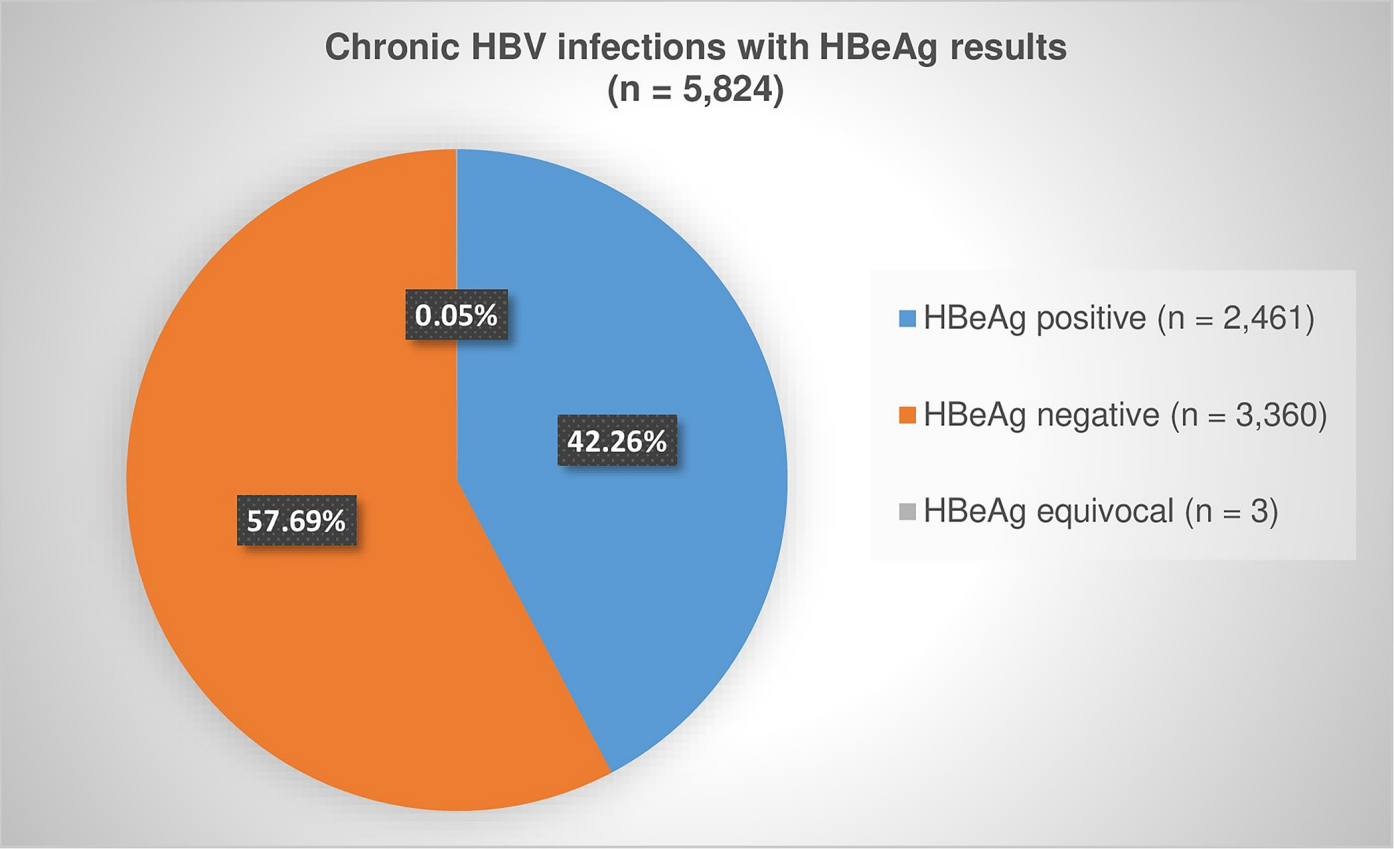
Proportion of chronic HBV infections with positive, negative and equivocal HBeAg results, 2015 to 2019.

Of the positive HBsAg dataset of 232,551 records, we identified 138,101 cases who had only a single positive test over the five-year period and 94,450 records of cases who had multiple positive HBsAg tests (Fig 1C). The 94,450 records were from 38,429 cases with at least two positive HBsAg tests, from which we identified 11,355 cases with samples collected 6 months or more apart (chronic HBV infections) (Fig 1C). One hundred and thirty five (135) cases tested HBsAg negative after being classified as chronic, whilst the majority (11,220) remained HBsAg positive up until the end of the study period (Fig 1C). Of the 11,355 chronic HBV infections, we linked 5,824 (51.29%) cases who had been tested for HBeAg (Table 4) with results illustrated in Fig 4.

**Table 4:** Distribution of chronic HBV infections by age group stratified by gender and HBeAg results, 2015 to 2019.

Age group	Gender	Chronic HBV		HBeAg					Age group	Gender	Chronic HBV		HBeAg				
		Positive		With results^b^		Positive^c^					Positive		With results^b^		Positive^c^		
		No.	*P-value* ^*a*^	No	%	No	%	*P-value* ^*a*^			No.	*P-value* ^*a*^	No	%	No	%	*P-value* ^*a*^
**0–4**	*Women*	3	*0*.*4924*	3	100	3	100	*0*.*3711*	**40–44**	*Women* ^*d*^	646	***<0*.*0001***	331	51.24	112	17.34	***<0*.*0001***
	*Men*	5		1	20.00	1	20.00			*Men*	1,131		546	48.28	228	20.16	
	*Unknown*	0		0	-	0	-			*Unknown*	14		5	35.71	4	28.57	
	***Total***	**8**		**4**	**50.00**	**4**	**50.00**			***Total***	**1,791**		**882**	**49.25**	**344**	**19.21**	
**5–9**	*Women*	5	*0*.*0640*	4	80.00	3	60.00	*0*.*2827*	**45–49**	*Women* ^*d*^	363	***<0*.*0001***	189	52.07	50	13.77	***<0*.*0001***
	*Men*	15		9	60.00	7	46.67			*Men*	757		381	50.33	137	18.10	
	*Unknown*	1		1	100	1	100			*Unknown*	13		10	76.92	4	30.77	
	***Total***	**21**		**14**	**66.67**	**11**	**52.38**			***Total***	**1,133**		**580**	**51.19**	**191**	**16.86**	
**10–14**	*Women*	11	*0*.*8547*	7	63.64	4	36.36	*>0*.*9999*	**50–54**	*Women*	224	***<0*.*0001***	125	55.80	21	9.38	***<0*.*0001***
	*Men*	10		7	70.00	4	40.00			*Men*	372		208	55.91	66	17.74	
	*Unknown*	0		0	-	0	-			*Unknown*	6		2	33.33	0	0.00	
	***Total***	**21**		**14**	**66.67**	**8**	**38.10**			***Total***	**602**		**335**	**55.65**	**87**	**14.45**	
**15–19**	*Women* ^*d*^	83	***<0*.*0001***	42	50.60	30	36.14	***0*.*0304***	**55–59**	*Women*	107	***<0*.*0001***	58	54.21	14	13.08	***0*.*0115***
	*Men*	31		23	74.19	15	48.39			*Men*	227		116	51.10	32	14.10	
	*Unknown*	0		0	-	0	-			*Unknown*	2		0	0.00	0	0.00	
	***Total***	**114**		**65**	**57.02**	**45**	**39.47**			***Total***	**336**		**174**	**51.79**	**46**	**13.69**	
**20–24**	*Women* ^*d*^	484	***<0*.*0001***	265	54.75	134	27.69	***<0*.*0001***	**60+**	*Women*	108	***0*.*0041***	76	70.37	18	16.67	*0*.*3006*
	*Men*	219		116	52.97	59	26.94			*Men*	155		92	59.35	25	16.13	
	*Unknown*	15		14	93.33	11	73.33			*Unknown*	5		2	40.00	1	20.00	
	***Total***	**718**		**395**	**55.01**	**204**	**28.41**			***Total***	**268**		**170**	**63.43**	**44**	**16.42**	
**25–29**	*Women* ^*d*^	1,021	***<0*.*0001***	501	49.07	277	27.13	***0*.*0001***	**Unknown**	*Women*	12	*0*.*0918*	9	75.00	2	16.67	*0*.*0577*
	*Men*	691		367	53.11	188	27.21			*Men*	23		18	78.26	10	43.48	
	*Unknown*	12		9	75.00	3	25.00			*Unknown*	2		0	0.00	0	0.00	
	***Total***	**1,724**		**877**	**50.87**	**468**	**27.15**			***Total***	**37**		**27**	**72.97**	**12**	**32.43**	
**30–34**	*Women* ^*d*^	1,064	***<0*.*0001***	475	44.64	204	19.17	***<0*.*0001***	**Total**	*Women*	5,027	***<0*.*0001***	2,527	50.27	1,046	20.81	***<0*.*0001***
	*Men*	1,272		683	53.69	338	26.57			*Men*	6,211		3,228	51.97	1,378	22.19	
	*Unknown*	23		11	47.83	8	34.78			*Unknown*	117		69	58.97	37	31.62	
	***Total***	**2,359**		**1,169**	**49.55**	**550**	**23.31**			***Total***	**11,355**		**5,824**	**51.29**	**2,461**	**21.67**	
**35-39**	*Women* ^*d*^	896	***<0*.*0001***	442	49.33	174	19.42	***<0*.*0001***									
	*Men*	1,303		661	50.73	268	20.57										
	*Unknown*	24		15	62.50	5	20.83										
	***Total***	**2,223**		**1,118**	**50.29**	**447**	**20.11**										

^a^P-value generated from the test of proportions between women and men, per age group and total

^b^Number and percentage of chronic HBV infections with available HBeAg results

^c^Number and percentage of chronic HBV infections with positive HBeAg results

^d^Women of reproductive age (15–49 years).

Of the total active HBV infections identified during the five-year period, 6.43% (11,355/176,530) were chronically infected. Of the total chronic HBV infections and those that were HBeAg positive, significantly more men tested positive than women (p < 0.0001), although at ages 15 to 29 years significantly more women tested positive than men (p ≤ 0.0339) (Table 4). Majority of the chronic HBV infections were among individuals aged 25 to 49 years old (9,230 / 11,355, 81.28%), and the majority of HBeAg positive chronic cases were among individuals aged 20 to 49 years old (2,204 / 2,461, 89.56%) (Table 4).

Among the 5,027 women with chronic HBV infection, 1,046 (20.81%) were HBeAg positive, and 4,557 (90.65%) and 981 (93.78%) were of reproductive age respectively. HBeAg positivity among chronic HBV infections in women of reproductive age was 21.53% (981/4,557) (Table 4).

**Fig 5:**
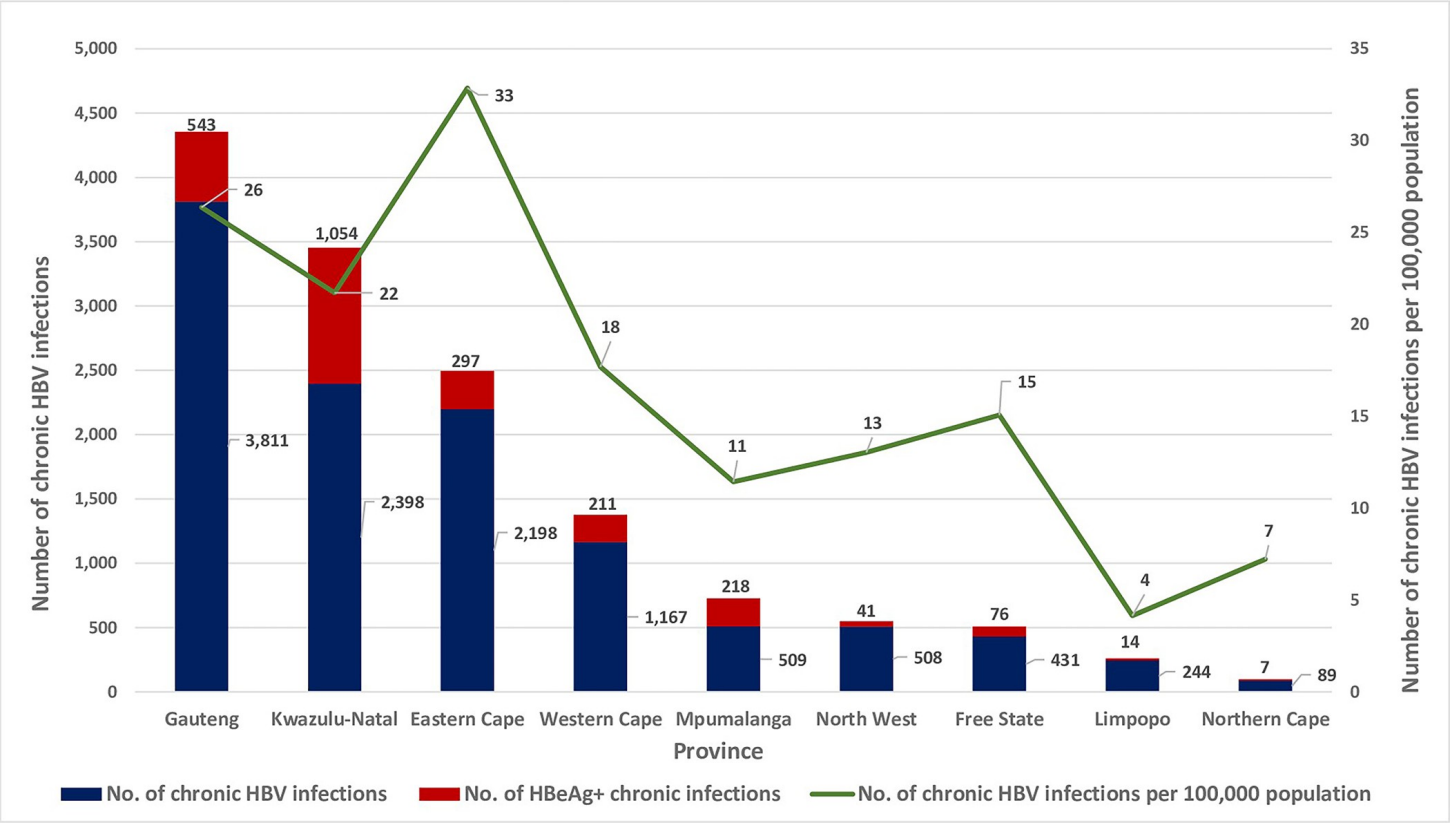
Provincial distribution of chronic HBV infections, those that were HBeAg positive, and chronic infections per 100,000 province population, 2015 to 2019.

At a provincial level, chronic HBV infection was highest in Gauteng (3,811, 33.56%), followed by Kwazulu-Natal (2,398, 21.12%) and Eastern Cape (2,198, 19.36%), with the lowest proportion in the Northern Cape (89, 0.78%) (Fig 5). Of the 2,461 HBeAg positive chronic cases, individuals from Kwazulu-Natal accounted for 42.83% of the cases (n = 1,054), individuals from Mpumalanga, Gauteng, Eastern Cape, Western Cape and Free State together accounted for 54.65% (n = 1,345) of the cases, and the remaining 2.52% of the cases (n = 62) were from North West, Limpopo and Northern Cape (Fig 5).

Chronic HBV infections per 100,000 province population was highest in Eastern Cape (33/100,000), followed by Gauteng (26/100,000) and Kwazulu-Natal (22/100,000), and least in Limpopo (4/100,000) (Fig 5).

By gender, chronicity was significantly more prevalent in men than women in six of the nine provinces (p ≤ 0.0254) (S3 Table), and HBeAg positivity among men was significantly higher than women in five of the nine provinces (p ≤ 0.0275) (S3 Table). In Eastern Cape, HBeAg positivity was higher in chronically infected men yet more women were found to have chronic infections. Contrarily, in Northern Cape, although numbers were low, HBeAg positivity was higher in chronically infected women yet more men were found to have chronic infections (S3 Table).

## 4 Summary and discussion

We analysed countrywide HBsAg and HBeAg laboratory data from 2015 to 2019 to determine the number of cases who had active HBV infection, including prolonged (chronic) infection, and their HBeAg status. Over the five years, the HBsAg test positivity rate was 9.02%, of which 6.43% were found to have chronic HBV infection. HBeAg test results were available for 37.13% of active infections and 51.29% among those with chronic infections, with test positivity rates of 37.89% and 42.26% respectively, indicating high levels of viral replication in these individuals, increased risk of HBV transmission, and an increased risk of progression to cirrhosis among those with chronic infections [24,30]. Despite the unavailability of HBeAg status for more than 60% of all active HBV infections and just under 50% of chronic infections, the positivity rate was surprisingly high at 14.07% and 21.67% respectively. The lack of HBeAg results may be due to patient loss to follow up, unawareness of or non-adherence to the testing algorithm, or HBeAg tests conducted outside of the study period.

We noted that the majority of active HBV infections were among cases who were born pre-1995, outside the vaccine-eligible era and therefore foreseeable. Active infections were considerably lower among individuals who were vaccine-eligible as infants (0 to 19 years), demonstrating effective vaccination in these groups. As vaccination is key to prevention of HBV infection, active HBV infections among vaccine-eligible individuals is likely due to the suboptimal vaccine coverage rates reported for South Africa from 2000 to 2019 [41–45]. Suboptimal vaccine coverage rates may have largely been due to the challenges that faced EPI activities in South Africa, highlighted by EPI managers as being insufficient knowledge of vaccines and EPI practices among staff, financial constraints, staff shortages and high staff turn-over [59].

HBV infection was seen to be more prevalent in men overall; however, at ages 10 to 29 years more women were infected. The higher rate among men overall is compounded by the fact that the average population size over the five years was higher in women. The higher rates observed in women 10 to 29 years old are likely due to their increased risk of symptomatic infection following viral infections, coupled with higher health seeking tendency including antenatal visits at these ages [60]. The higher rates in men overall and at later ages are likely due to their increased risk of persistent HBV infection resulting in their increased risk of HBV chronicity [61,62]. This is in keeping with studies conducted in Taiwan, Greece and New Zealand, where it was reported that women were less likely to develop chronic HBV infection [62–64]. In a study on sex differences in response to HBV infection, Blumberg reports that the prevalence of chronic infection among men is higher than women in most human populations [65].

In children under five years old, we noted a sizeable number of active HBV infections over the five years (1,131). Although horizontal transmission has been reported as a major transmission route between children in South Africa, justifiable reasons are lacking [36,66]. Looking closer into these children, 65.78% were less than one year old. With no birth dose of the HBV vaccine administered to these children and no routine screening for HBV infection at antenatal care, we suspect a considerable proportion were infected from their mothers given the increased risk with no preventative interventions [6,15,39,40]. Additionally, 6.90% (78) were HBeAg positive. Honing in, we saw that 34.54% of children under one year old were at ages below the first scheduled HBV vaccine dose at 6 weeks of age and 19.09% were at ages post two weeks after the third scheduled HBV vaccine dose at 14 weeks of age, eliminating transient HBsAg positivity following vaccination if the vaccine was administered as per the EPI schedule at 6, 10 and 14 weeks of age [67]. Furthermore, our analyses of women alone showed that of the total with active HBV infections, 85.17% were of reproductive age with an HBeAg prevalence of 13.73%, indicative of an increased risk of mother-to-child transmission [33,34]. Considering the WHO impact target of ≤0.1% HBsAg prevalence for measuring elimination in children under five years old and the South African HBsAg population positivity rate in this age group (population prevalence), South Africa is well below the target over 2015 to 2019 at 0.02% [22]. However, the data presented are only from children tested in public healthcare facilities and it is likely that a substantial proportion of children under five years old were not HBV diagnosed given their low risk of symptomatic infection [1]. Substituting the denominator with the total number of cases under five years old who were tested for HBsAg, the HBsAg test positivity rate (test prevalence) was 4.83%, well above the WHO impact target of ≤0.1%.

Provincially, we noted that the majority of active HBV infections were in Gauteng, followed by Kwazulu-Natal and Eastern Cape and lowest in the Northern Cape, not surprising given that the average population size of these four provinces from 2015 to 2019 followed the same pattern. In addition, the findings in Gauteng and Northern Cape may also be related to the land size of these provinces versus their population sizes [68]. Furthermore, Northern Cape boasted the highest average vaccine coverage rate from 2000 to 2019 (85.57%) (S1 Table). Focussing on HBeAg positivity, we saw that more than 50% of cases were from Kwazulu-Natal. Similarly, we noted that of all HBeAg results available, 51.60% were from Kwazulu-Natal (Table 2). This finding suggests that in Kwazulu-Natal, follow up testing was considerably more prevalent than in other provinces over the study period, likely related to the testing facility and testing practises. Taking into consideration the population size per province, Eastern Cape was seen to have the highest number of chronic HBV infections in the country (33/100,000), in keeping with one of the poorest vaccine coverage rates averaging 69,54% from 2000 to 2019 (S1 Table). Surprisingly, however, there were more chronic infections in women in Eastern Cape, despite significantly higher active infections in men. Regarding gender distribution, we saw that active HBV infections were significantly higher in women in Limpopo province, yet more men were chronically infected. We link this finding in Limpopo to the increased risk of symptomatic infection following viral infections in women together with the increased risk of persistent HBV infection in men [60–62]. Although reasons are unclear, we are cognisant of provincial public health disparities within South Africa and suboptimal vaccine coverage rates (S1 Table) [69]. Therefore, provincial investigations, including community awareness, HBV testing and follow up practises, social practises, and vaccination policies would provide insight into provincial HBV burden.

From analyses of cases who had both positive and negative HBsAg results over the five years, we saw that 64.42% (5,569/8,645) of cases tested negative after a positive test, suggesting HBsAg clearance and resolution of HBV infection. Analyses of testing patterns showed that among the cases who tested negative after a positive test, 11.92% (664/5,569) of cases tested negative before testing positive, suggesting that in these individuals HBV infection was likely acquired during the study period and likely among those individuals who did not receive or complete their primary course of vaccinations. Similarly for cases who tested HBsAg negative initially and positive subsequently (3,003/8,645, 34.74%). We also observed a small proportion (73/8,645, 0.84%) of cases who tested HBsAg negative between two positive tests, suggesting HBsAg clearance after acquiring HBV infection followed by reactivation of HBV disease or HBV reinfection, a phenomenon seen among HIV-1 infected individuals [8]. Among the 11,355 chronic HBV infections, we report a last negative HBsAg test in 1.19% of cases following classification as chronic, in line with reported statistics of HBsAg clearance observed in 1–2% of chronic infections following the natural course of disease or treatment interventions [13,14].

A limitation of this study is that we report findings restricted to passive surveillance of HBsAg and HBeAg tests conducted over a five-year fixed period. Population prevalence rates include South Africa’s total population as the denominator and our findings are from individuals attending only public healthcare facilities. HBeAg results were available for less than 50% of active infections. We noted a large number of cases who tested positive for HBsAg repeatedly but sample collection dates fell short of 6 months apart. Our numbers, therefore, represent minimum estimates. We analysed data on women of reproductive age as a proxy of pregnant women. HBsAg positivity among children 6 to 16 weeks of age may be associated with transient positivity following vaccination rather than active HBV infection, although reasons for testing in this age group are unclear and vaccination data was not available. Although we are cognisant of the possibility of false results, we did not factor this in during our analyses. Our data were not linked to HIV status and we therefore cannot report on HBV and HIV co-infection or relate our findings to other studies reporting HBV prevalence and chronicity in HIV-infected individuals.

A strength of our study, however, is the interrogation of a robust nationally representative public health dataset comprising over 2,3 million HBsAg records and 100 000 HBeAg records.

## 5 Conclusion

Prevention of HBV infection through infant vaccination, with implementation of a birth dose, is key to eliminating HBV infection. Given that the HBV vaccine is administered as a hexavalent vaccine in South Africa, optimal (3-dose) HBV vaccine coverage would not only reduce HBV burden, but would provide protection from other debilitating vaccine-preventable diseases. Routine screening of pregnant women attending antenatal clinics for HBV and appropriate maternal antiviral therapy would further reduce mother-to-child transmission. Actual data on the national prevalence of HBV in pregnant women is needed to inform policy. Early identification of HBV chronicity is fundamental in reducing the risk of liver cirrhosis and hepatocellular carcinoma through appropriate treatment initiation, and can be achieved through real-time data analysis and dissemination of the relevant information for public health interventions.

## Supporting information

S1 Table Vaccination coverage among children under one year who have completed their primary course of immunisation in South Africa. (PDF)

S2 Table Active HBV infections by province stratified by genger and HBeAg results, 2015 to 2019. (PDF)

S3 Table Chronic HBV infections by province stratified by genger and HBeAg results, 2015 to 2019. (PDF)

S1 Dataset (ZIP)

S2 Dataset (ZIP)

S3 Dataset (ZIP)

S4 Dataset (ZIP)
