# Supplementary material for: Hepatitis B infection status among South Africans attending public health facilities over a five-year period: 2015 to 2019
Source: PLOS Glob Public Health. 2023 Sep 25;3(9):e0000992. doi: 10.1371/journal.pgph.0000992 (PMC10519597; doi:10.1371/journal.pgph.0000992)
Supplement: S1 Table — (PDF) [file pgph.0000992.s001.pdf]

S1 Table: Vaccination coverage amongst children under one year who have completed their primary course of immunisation in South Africa*

Province	2000	2001	2002	2003	2004	2005	2006	2007	2008	2009	2010	2011	2012	2013	2014	2015	2016	2017	2018	2019	Average (2000-2019)
**Eastern Cape**	68.21	64.51	67.03	72.68	72.96	76.75	74.34	74.54	64.80	70.50	60.30	65.30	67.20	66.50	71.90	73.10	63.90	68.40	71.90	76.00	**69.54**
**Free State**	70.44	67.96	68.16	69.53	87.08	102.20	109.42	109.07	93.40	90.00	81.30	84.30	84.60	77.80	80.80	72.70	68.50	71.20	74.80	77.40	**82.03**
**Gauteng**	66.16	64.70	66.54	72.22	76.93	92.28	98.58	101.06	85.40	90.20	91.50	98.60	97.20	98.20	94.10	86.60	74.70	76.80	84.40	86.90	**85.15**
**Kwazulu-Natal**	72.83	68.82	69.55	70.02	71.21	72.07	79.86	77.82	64.20	65.20	64.60	75.60	74.90	76.20	80.10	74.40	74.00	81.30	90.80	91.40	**74.74**
**Limpopo**	77.77	72.47	77.54	77.08	83.27	89.61	93.21	84.15	79.70	82.10	71.60	70.60	68.60	68.90	79.70	76.30	60.00	70.40	71.00	73.60	**76.38**
**Mpumalanga**	65.38	62.14	62.88	66.47	68.86	76.05	77.63	78.49	65.80	71.40	54.30	58.90	69.20	74.40	81.50	85.90	75.60	89.70	96.80	96.60	**73.90**
**North West**	71.09	66.57	59.43	63.49	64.28	71.83	68.39	76.61	67.50	64.50	56.60	59.60	64.40	67.30	76.50	76.20	69.00	69.40	68.40	63.00	**67.20**
**Northern Cape**	66.85	67.94	67.70	70.92	81.20	89.06	95.90	93.31	87.30	82.90	83.10	90.50	94.20	94.30	98.90	96.20	90.80	83.90	87.50	89.00	**85.57**
**Western Cape**	82.22	68.56	73.69	76.53	76.03	80.63	87.49	87.62	94.70	94.80	82.00	81.40	82.50	80.40	87.10	84.90	74.80	80.90	82.70	84.90	**82.19**
**South Africa**	**71.22**	**67.07**	**68.06**	**70.99**	**75.76**	**83.39**	**87.20**	**86.96**	**78.09**	**79.07**	**71.70**	**76.09**	**78.09**	**78.22**	**83.40**	**80.70**	**72.37**	**76.89**	**80.92**	**82.09**	**77.41**

**As published by the Health Systems Trust of South Africa from data obtained from the District Health Information System*
