# Supplementary material for: Hepatitis B infection status among South Africans attending public health facilities over a five-year period: 2015 to 2019
Source: PLOS Glob Public Health. 2023 Sep 25;3(9):e0000992. doi: 10.1371/journal.pgph.0000992 (PMC10519597; doi:10.1371/journal.pgph.0000992)
Supplement: S2 Table — (PDF) [file pgph.0000992.s002.pdf]

S2 Table: Active HBV infections by province stratified by genger and HBeAg results, 2015 to 2019

Province		Gender				
		Women (No.)	Men (No.)	Unknown (No.)	Total (No.)	*P Value* [a]
	**Eastern Cape**					
	Active HBV infections	10,368	10,685	227	21,280	***0.0306***
	HBeAg +	1,116	1,272	24	2,412	***0.0016***
	HBeAg -	2,476	2,540	45	5,061	-
	HBeAg eq [b]	0	0	0	0	-
	HBeAg null [c]	6,776	6,873	158	13,807	-
	**Free State**					
	Active HBV infections	3,593	4,447	47	8,087	***<0.0001***
	HBeAg +	425	672	3	1,100	***<0.0001***
	HBeAg -	370	482	2	854	-
	HBeAg eq [b]	4	9	0	13	-
	HBeAg null [c]	2,794	3,284	42	6,120	-
	**Gauteng**					
	Active HBV infections	28,547	35,481	1,057	65,085	***<0.0001***
	HBeAg +	1,067	1,676	52	2,795	***<0.0001***
	HBeAg -	1,546	2,069	57	3,672	-
	HBeAg eq [b]	1	0	0	1	-
	HBeAg null [c]	25,933	31,736	948	58,617	-
	**Kwazulu-Natal**					
	HBsAg+	15,165	19,262	1,105	35,532	***<0.0001***
	Active HBV infections	5,485	7,440	407	13,332	***<0.0001***
	HBeAg -	8,813	11,019	643	20,475	-
	HBeAg eq [b]	11	6	0	17	-
	HBeAg null [c]	856	797	55	1,708	-
	**Limpopo**					
	Active HBV infections	5,153	4,894	75	10,122	***0.0103***
	HBeAg +	142	153	4	299	*0.5276*
	HBeAg -	242	276	2	520	-
	HBeAg eq [b]	0	0	0	0	-
	HBeAg null [c]	4,769	4,465	69	9,303	-
	**Mpumalanga**					
	Active HBV infections	6,768	7,235	173	14,176	***0.0001***
	HBeAg +	1,309	1,909	32	3,250	***<0.0001***
	HBeAg -	3,466	3,198	85	6,749	-
	HBeAg eq [b]	0	0	0	0	-
	HBeAg null [c]	1,993	2,128	56	4,177	-
	**North West**					
	Active HBV infections	4,153	5,255	132	9,540	***<0.0001***
	HBeAg +	137	228	1	366	***<0.0001***
	HBeAg -	256	278	4	538	-
	HBeAg eq [b]	2	1	0	3	-
	HBeAg null [c]	3,758	4,748	127	8,633	-
	**Northern Cape**					
	Active HBV infections	806	993	28	1,827	***<0.0001***
	HBeAg +	22	25	1	48	*0.6689*
	HBeAg -	45	62	0	107	-
	HBeAg eq [b]	0	1	0	1	-
	HBeAg null [c]	739	905	27	1,671	-
	**Western Cape**					
	Active HBV infections	4,381	6,446	54	10,881	***<0.0001***
	HBeAg +	453	782	2	1,237	***<0.0001***
	HBeAg -	1,042	1,649	4	2,695	-
	HBeAg eq [b]	2	4	0	6	-
	HBeAg null [c]	2,884	4,011	48	6,943	-
	**Total**					
	Active HBV infections	78,935	94,699	2,896	176,530	***<0.0001***
	HBeAg +	10,156	14,157	526	24,839	***<0.0001***
	HBeAg -	18,256	21,573	842	40,671	-
	HBeAg eq [b]	20	21	0	41	-
	HBeAg null [c]	50,503	58,948	1,528	110,979	-

[a] *P value generated from the test of proportions between women and men, total and per province*

[b] *Equivocal (inconclusive) HBeAg results*

[c] *No HBeAg results available*
