# Supplementary material for: Hepatitis B infection status among South Africans attending public health facilities over a five-year period: 2015 to 2019
Source: PLOS Glob Public Health. 2023 Sep 25;3(9):e0000992. doi: 10.1371/journal.pgph.0000992 (PMC10519597; doi:10.1371/journal.pgph.0000992)
Supplement: S3 Table — (PDF) [file pgph.0000992.s003.pdf]

S3 Table: Chronic HBV infections by province stratified by genger and HBeAg results, 2015 to 2019

Province	Women (No.)	Men (No.)	Unknown (No.)	Total (No.)	*P Value* [a]
**Eastern Cape**					
Chronic HBV infections	1,133	1,060	5	2,198	*0.0612*
HBeAg +	142	155	0	297	*0.4910*
HBeAg -	280	249	1	530	
HBeAg eq [b]	0	0	0	0	
HBeAg null [c]	711	656	4	1,371	
**Free State**					
Chronic HBV infections	166	265	0	431	***<0.0001***
HBeAg +	28	48	0	76	***0.0284***
HBeAg -	30	56	0	86	
HBeAg eq [b]	0	1	0	1	
HBeAg null [c]	108	160	0	268	
**Gauteng**					
Chronic HBV infections	1,626	2,146	39	3,811	***<0.0001***
HBeAg +	233	307	3	543	***0.0013***
HBeAg -	243	349	4	596	
HBeAg eq [b]	0	0	0	0	
HBeAg null [c]	1,150	1,490	32	2,672	
**Kwazulu-Natal**					
Chronic HBV infections	1,016	1,325	57	2,398	***<0.0001***
HBeAg +	438	585	31	1,054	***<0.0001***
HBeAg -	572	735	26	1,333	
HBeAg eq [b]	1	1	0	2	
HBeAg null [c]	5	4	0	9	
**Limpopo**					
Chronic HBV infections	115	125	4	244	*0.5358*
HBeAg +	5	9	0	14	*0.3138*
HBeAg -	18	25	0	43	
HBeAg eq [b]	0	0	0	0	
HBeAg null [c]	92	91	4	187	
**Mpumalanga**					
Chronic HBV infections	227	280	2	509	***0.0251***
HBeAg +	92	125	1	218	***0.0290***
HBeAg -	115	127	0	242	
HBeAg eq [b]	0	0	0	0	
HBeAg null [c]	20	28	1	49	
**North West**					
Chronic HBV infections	225	276	7	508	***0.0260***
HBeAg +	19	22	0	41	*0.6094*
HBeAg -	29	28	1	58	
HBeAg eq [b]	0	0	0	0	
HBeAg null [c]	177	226	6	409	
**Northern Cape**					
Chronic HBV infections	36	52	1	89	*0.0968*
HBeAg +	4	2	1	7	*0.5174*
HBeAg -	5	8	0	13	
HBeAg eq [b]	0	0	0	0	
HBeAg null [c]	27	42	0	69	
**Western Cape**					
Chronic HBV infections	483	682	2	1,167	***<0.0001***
HBeAg +	85	125	1	211	***0.0069***
HBeAg -	188	271	0	459	
HBeAg eq [b]	0	0	0	0	
HBeAg null [c]	210	286	1	497	
**Total**					
Chronic HBV infections	5,027	6,211	117	11,355	***<0.0001***
HBeAg +	1,046	1,378	37	2,461	***<0.0001***
HBeAg -	1,480	1,848	32	3,360	
HBeAg eq [b]	1	2	0	3	
HBeAg null [c]	2,500	2,983	48	5,531	

Gender columns: Women (No.), Men (No.), Unknown (No.), Total (No.)

[a] *P value generated from the test of proportions between women and men, per province and total*

[b] *Equivocal (inconclusice) HBeAg results*

[c] *No HBeAg results available*
